# An Uncommon Extensive Bilateral Continuous Ossification of the Stylohyoid Chain Extending to the Hyoid Bone in Eagle’s Syndrome: A Case Report

**DOI:** 10.7759/cureus.107768

**Published:** 2026-04-26

**Authors:** Garima Thapa, Wu Xian Tao, Lv Zhong Jing, Yuan Jian

**Affiliations:** 1 Department of Oral and Maxillofacial Surgery, The Affiliated Hospital of Xuzhou Medical University, Xuzhou, CHN

**Keywords:** cbct, eagle’s syndrome, elongated styloid process, extra oral approach, stylohyoid complex

## Abstract

Eagle’s syndrome is a rare cause of cervicofacial pain and dysphagia arising from elongation of the styloid process or ossification of the stylohyoid ligament complex. While styloid elongation >3 cm is relatively common, extensive continuous ossification of the stylohyoid chain with continuity to the hyoid bone remains uncommon.

We report a 42-year-old man with a six-month history of persistent throat pain and globus sensation unresponsive to conservative measures. Clinical examination showed bilateral tonsillar fossa tenderness with palpable bony projections, more pronounced on the left. Cone-beam computed tomography (CBCT) revealed marked bilateral elongation and ossification of the stylohyoid complex, measuring 8.04 cm on the right and 10.11 cm on the left, exceeding the longest previously reported length of 8.5 cm. Due to the extent and proximity to the hyoid bone, surgical excision was performed via an extraoral transcervical approach, resulting in complete symptom resolution.

This case represents one of the most extensive bilateral ossifications of the stylohyoid chain documented, with the longest reported stylohyoid complex to date. Such extreme elongation likely contributed to mechanical irritation and restricted neck movement. Early recognition with advanced imaging (CBCT) and tailored surgical planning are critical to avoid misdiagnosis and achieve excellent outcomes in these rare variants.

## Introduction

The condition now known as Eagle’s syndrome was first described by Eagle in 1937, who reported patients presenting with pharyngeal irritation and orofacial pain associated with an elongated styloid process [1]. The styloid process is a thin, pointed bony projection arising from the temporal bone that serves as an attachment for the stylohyoid ligament and several muscles involved in swallowing and phonation. It typically measures 2.5-3 cm in length, and elongation beyond 3 cm is generally considered abnormal [1,2]. Its close anatomical relationship with major neurovascular structures, including the internal carotid artery, internal jugular vein, and cranial nerves IX, X, XI, and XII, explains the diverse symptomatic presentations observed in Eagle’s syndrome [3]. The prevalence of styloid process elongation in radiographic studies has been reported at approximately 4%-7% of the population, while only a small proportion (about 4%-10%) are symptomatic, resulting in clinically significant Eagle’s syndrome being relatively rare [3-5]. The condition is reported to occur more frequently in women and typically presents in the fourth decade of life [5,6]. Clinical manifestations of Eagle’s syndrome may include throat pain, dysphagia, foreign body sensation in the pharynx, otalgia, and restricted neck movements [5]. Because of its variable and nonspecific clinical presentation, Eagle’s syndrome is frequently misdiagnosed as temporomandibular joint disorders, cranial neuralgias (such as glossopharyngeal or trigeminal neuralgia), chronic pharyngeal infections, otologic conditions, dental pathologies, or cervical spine disorders [6,7]. Therefore, careful clinical evaluation combined with appropriate radiographic imaging, including panoramic radiography, computed tomography (CT), and cone-beam computed tomography (CBCT), is essential for establishing an accurate diagnosis [6].

Although bilateral cases with ossification of the stylohyoid chain have been reported, extensive continuous ossification reaching or articulating with the hyoid bone remains rare. Reports describing such extreme elongation with uninterrupted ossified continuity to the hyoid are exceptionally limited in the literature. The present report describes a rare case of bilateral Eagle’s syndrome with marked ossification of the stylohyoid ligament complex extending to and articulating with the hyoid bone, including a left-sided measurement of 10.11 cm, representing the longest documented stylohyoid complex to date.

## Case presentation

A 42-year-old man presented to the Department of Oral and Maxillofacial Surgery with a six-month history of persistent dull throat pain, globus sensation, and occasional headaches. The pain was intermittent, of mild-to-moderate intensity, and aggravated by swallowing. He reported restricted neck movements, particularly difficulty turning his head to the left. Pain severity was assessed clinically based on patient-reported intensity and functional limitation; however, no standardized pain scoring system (such as visual analog score (VAS) or numerical rating scale (NRS)) was used. The patient had a known history of hypertension managed with regular medication and had received multiple courses of antibiotics for presumed pharyngitis without symptom relief.

Extraoral examination revealed normal facial symmetry with no palpable cervical or submandibular masses and no tenderness over the muscles of mastication. Temporomandibular joint examination was unremarkable, with normal mandibular movements and no tenderness. Intraoral palpation of the tonsillar fossae elicited bilateral tenderness and revealed palpable bony projections, more prominent on the left side. Differential diagnoses, including temporomandibular joint disorders, glossopharyngeal neuralgia, chronic tonsillitis, and cervical spine-related pain, were considered and excluded based on clinical examination and radiological findings.

**Figure 1:**
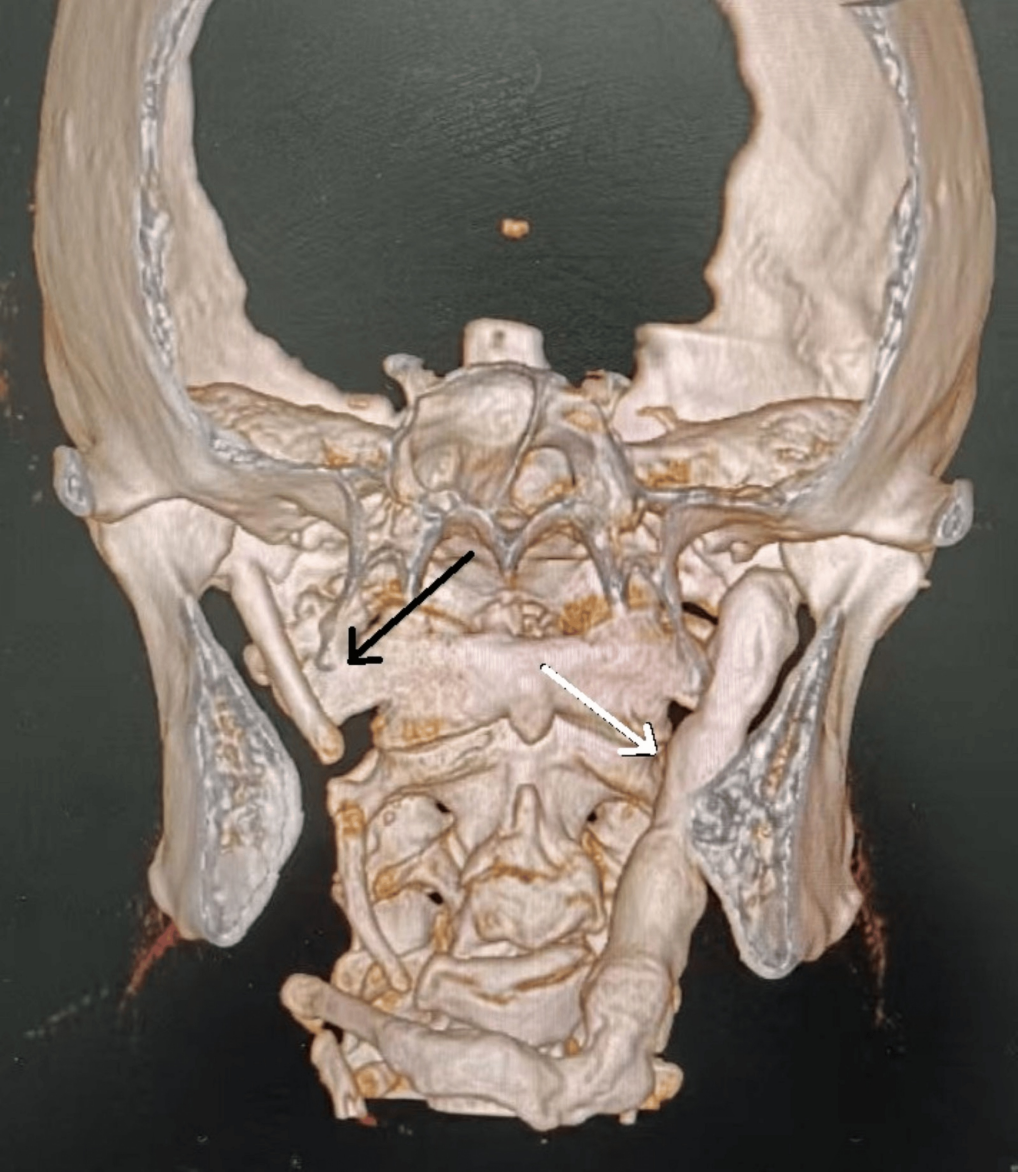
3D Cone-beam computed tomography (CBCT) reconstruction showing bilateral elongation of the styloid processes, right (black arrow) and left side (white arrow).

**Figure 2:**
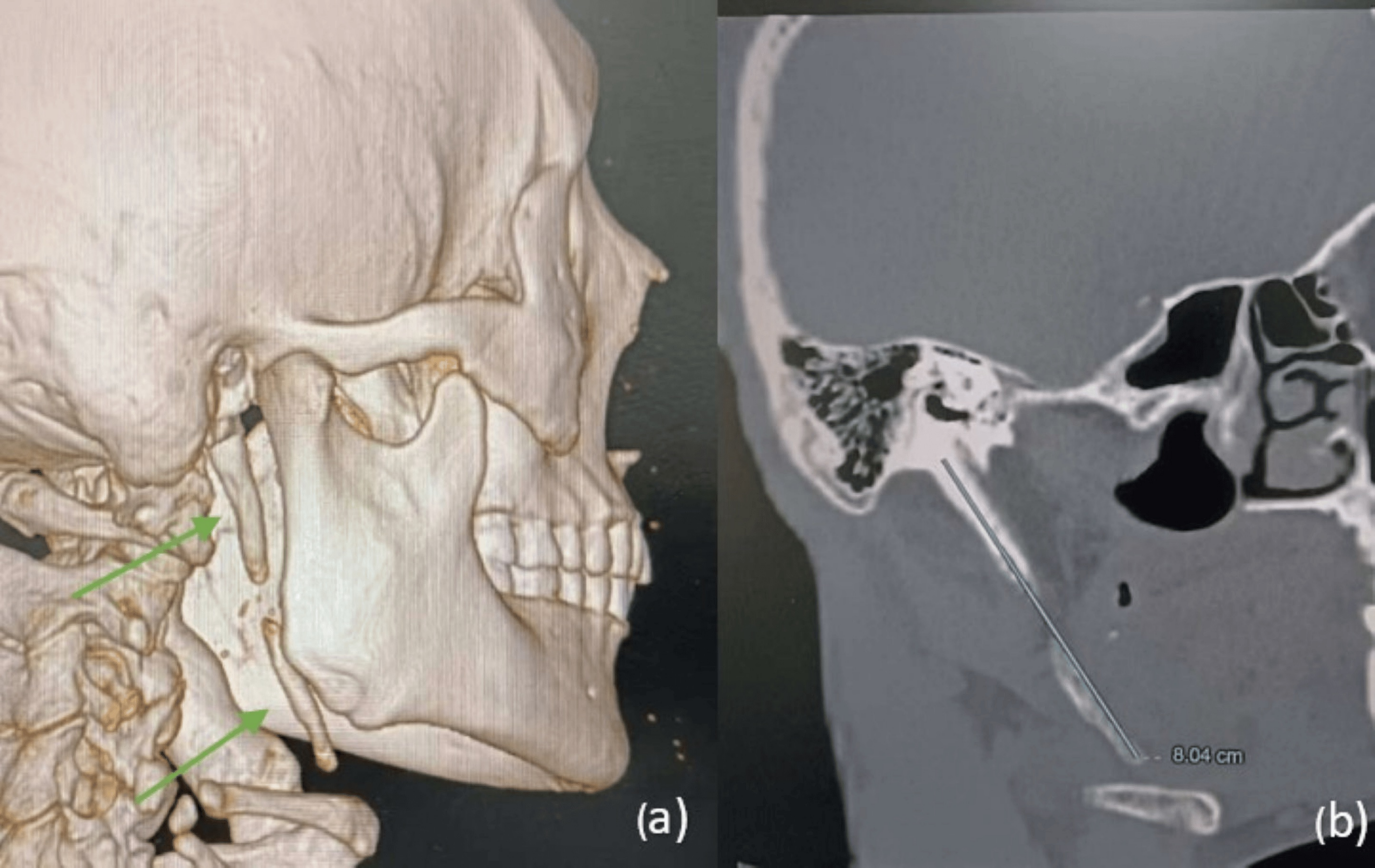
(a) 3D-reconstructed view of the right styloid process; (b) Sagittal cone-beam computed tomography (CBCT) slice showing measurement of the right styloid–stylohyoid complex (8.04 cm)

**Figure 3:**
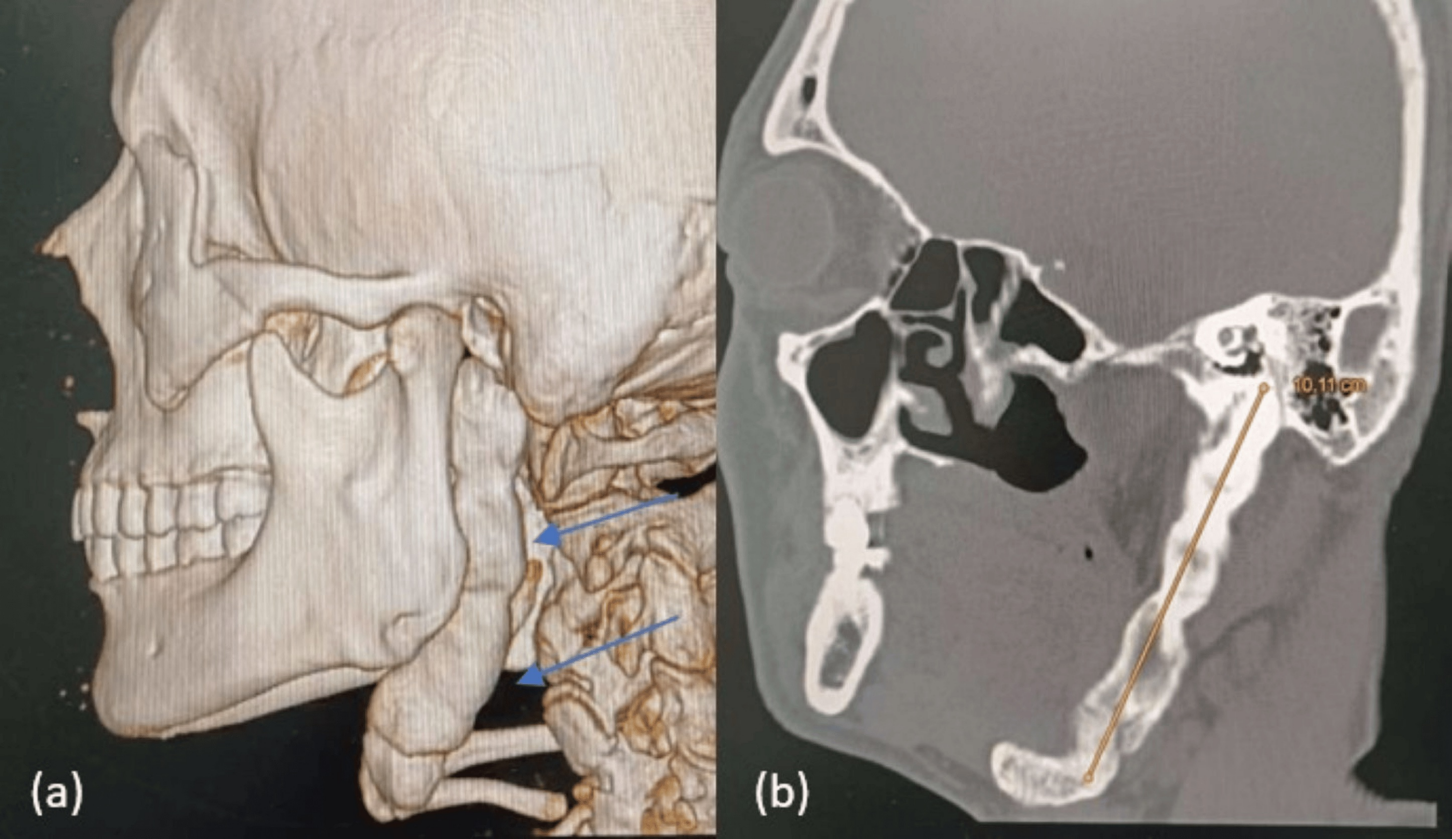
(a) 3D- reconstructed view of the left styloid process; (b) Sagittal cone-beam computed tomography (CBCT) slice showing measurement of the left styloid–stylohyoid complex (10.11 cm)

Cone-beam computed tomography (CBCT) with 3D reconstruction was performed to evaluate the stylohyoid complex and its relationship to adjacent structures. Imaging demonstrated bilateral elongation and ossification of the stylohyoid complex (Figure 1). The right side showed an elongated styloid process with segmental calcification of the stylohyoid ligament, and the left side exhibited a continuous ossified stylohyoid chain extending inferiorly with continuity to the hyoid bone. Measurements along the long axis in sagittal sections revealed a right-sided length of 8.04 cm (Figure 2) and a left-sided length of 10.11 cm (Figure 3). The length was measured from the base of the styloid process (temporal bone) to the distal end of the ossified ligament. The diagnosis was based on a combination of detailed clinical examination and high-resolution CBCT imaging with 3D reconstruction, allowing precise morphological assessment and linear measurement of the stylohyoid complex.

The three-dimensional (3D) reconstructions described in the figures were generated from CT Digital Imaging and Communications in Medicine (DICOM) datasets acquired on a multi-slice CT scanner and processed using the institutional picture archiving and communication system (PACS). Post-processing and visualization were performed using a web-based DICOM viewer (Universal Viewer, Zero-Footprint client), integrated within the hospital radiology information system at the Affiliated Hospital of Xuzhou Medical University (Xuzhou, China). The 3D reconstructions were obtained using volume rendering techniques with multiplanar reconstruction (MPR) for anatomical assessment.

Surgical management was planned via an extraoral transcervical approach under general anesthesia with nasotracheal intubation. A curvilinear incision was made in the left upper neck at the level of the hyoid bone. After elevation of skin, subcutaneous tissue, and platysma, careful dissection preserved adjacent neurovascular structures. The ossified stylohyoid chain was exposed, and the segment extending to the hyoid bone was resected using bone rongeurs, followed by removal of the distal ossified portions and smoothing of sharp bone edges. Intraoperative assessment confirmed improved head and neck mobility. Hemostasis was achieved, a negative-pressure drain was placed, and the wound was closed in layers.

The postoperative course was uneventful. The patient was discharged on the third postoperative day. Mild discomfort resolved within one week, and he remained completely symptom-free at the one-month follow-up, with full resolution of throat pain, globus sensation, and restricted neck movement. The patient expressed high satisfaction with the outcome.

## Discussion

This case illustrates bilateral Eagle’s syndrome with exceptionally extensive ossification of the stylohyoid complex. The left-sided measurement of 10.11 cm represents, to the best of our knowledge, the longest documented stylohyoid chain in the literature, surpassing the previous maximum of 8.5 cm reported to date [8]. The continuous ossification extending to the hyoid bone bilaterally is also uncommon, as most reported cases show only partial elongation or segmental calcification.

The exact etiology of Eagle’s syndrome remains uncertain. Several theories have been proposed, including congenital elongation of the styloid process, reactive ossification of the stylohyoid ligament following trauma or surgical procedures such as tonsillectomy, and age-related degenerative changes leading to ligament calcification [9]. These processes may result in elongation of the styloid process or ossification of the stylohyoid ligament complex, which may irritate adjacent neurovascular structures and produce the characteristic symptoms of Eagle’s syndrome [6,9]. The markedly elongated and ossified stylohyoid chains likely produced mechanical irritation or compression of adjacent structures, particularly the glossopharyngeal nerve and surrounding soft tissues. This is supported by preoperative findings of tonsillar fossa tenderness, globus sensation, throat pain aggravated by swallowing, and restricted left-sided neck rotation that resolved completely within one week postoperatively and remained absent at one-month follow-up. The rapid and sustained improvement without additional therapy provides strong evidence for a mechanical etiology in this extreme presentation. It helps differentiate it from alternative diagnoses such as chronic pharyngitis, temporomandibular joint disorder, or idiopathic neuralgia (which failed to respond to antibiotics and showed no temporomandibular disorders (TMJ) abnormalities on examination). Although key differential diagnoses such as glossopharyngeal neuralgia, temporomandibular disorders, and tonsillitis were considered, detailed exclusion was not comprehensively documented.

Similar cases of extensive stylohyoid ossification have been reported in the literature, although most demonstrate shorter segments or unilateral involvement. Previous reports by Panwar et al. [10] and Bijai et al. [11] described bilateral elongation; however, continuous ossification extending to the hyoid bone remains rarely documented. The comparison of some reported cases is shown in Table 1. Several radiographic classifications have been proposed to describe the elongation of the styloid process, the most widely used being the Langlais classification [12]. According to Langlais et al., this case revealed a Type III (segmented) pattern on the right and a Type I (continuous elongated) pattern on the left, further illustrating morphological heterogeneity even within the same patient [12]. CBCT with 3D reconstruction proved superior for precise length measurement, morphological assessment, and surgical planning, particularly given the proximity of the ossified segments to the hyoid bone and neurovascular structures [13,14].

**Table 1:** Comparison of selected reported cases of extensive or bilateral Eagle’s syndrome with stylohyoid ossification

Study	Laterality	Longest Length	Hyoid Extension / Continuity	Surgical Approach	Outcome
Present case (2026)	Bilateral	10.11 cm (left)	Yes	Extraoral transcervical	Complete resolution at one month
Anuradha et al. [7]	Bilateral	Not mentioned	Not reported	Extra-oral	Symptom-free after 24 hours
Alaqeeli et al. [8]	Unilateral	8.5 cm	Not reported	Not specified	Successful surgical treatment
Panwar et al. [10]	Bilateral	7.074 cm	Not reported	Transoral	Symptom-free at 3 months

The management of Eagle’s syndrome ranges from conservative (analgesics, anti-inflammatories, steroid injections) to surgical excision. Non-surgical treatment with various medications such as acetaminophen, tramadol, gabapentin, ganglion block, and local anesthetic injection has also been reported [14,15]. A comparative case report by Aravindan et al. demonstrated that both intraoral and extraoral approaches are effective for styloidectomy, with the transcervical approach providing superior surgical access in complex cases. In contrast, the transoral approach avoids external scarring [16]. In this case, the extraoral transcervical approach was chosen due to the extreme length, bilateral involvement, and distal extension to the hyoid, providing excellent visualization, safe neurovascular dissection, and the ability to assess head mobility intraoperatively. Accurate CBCT measurements with 3D reconstruction, strong clinical-radiological correlation, intraoperative assessment of head mobility, and rapid postoperative symptom relief after resection of an extremely elongated ossified chain highlight the significance of this case. Collectively, these aspects reinforce the educational relevance of this case, emphasizing the importance of advanced imaging modalities and individualized surgical approaches in managing rare anatomical variants. Despite being a rare anatomical variant, recognition of extensive stylohyoid ossification is essential to avoid misdiagnosis and unnecessary treatment for chronic orofacial pain conditions. Limitations include the single-case design and relatively short follow-up (one month), which limits assessment of long-term outcomes and recurrence. Furthermore, pain severity in this case was assessed clinically and not quantified using a standardized pain scoring system, which may limit objective assessment. Despite these limitations, this case provides valuable insights into the management of extreme stylohyoid chain ossification.

## Conclusions

This report describes bilateral Eagle’s syndrome featuring the longest documented stylohyoid complex (10.11 cm) with extensive ossification extending toward the hyoid bone. It underscores the importance of including Eagle’s syndrome in the differential diagnosis of persistent throat pain and globus sensation when common causes are excluded. Advanced imaging, particularly CBCT with 3D reconstruction, is indispensable for accurate diagnosis, morphological classification, and precise measurement in extreme cases. Surgical excision via an extraoral transcervical approach can yield rapid and complete symptom relief with low morbidity, as evidenced by the mechanical nature of symptoms in this patient. Early recognition and individualized surgical planning lead to excellent clinical outcomes even in unusually extensive presentations.
